# Clinical efficacy and acceptability of panretinal photocoagulation combined with conbercept for patients with proliferative diabetic retinopathy: A protocol for systematic review and meta-analysis

**DOI:** 10.1097/MD.0000000000025611

**Published:** 2021-04-30

**Authors:** Liangyu Wang, Zhaoli Chen, Xiaoxue Wang

**Affiliations:** a Department of Ophthalmology, Qingdao Municipal Hospital (Group); b Department of Ophthalmology, Qingdao Aier Eye Hospital, Shandong, China.

**Keywords:** conbercept, meta-analysis, panretinal photocoagulation, proliferative diabetic retinopathy, protocol

## Abstract

**Background:**

Although conbercept has been used for other diseases associated with new vascular formation, the effect of single-dose conbercept in combination with proliferative diabetic retinopathy (PDR) have not been established. We thus conducted this protocol for systematic review and meta-analysis to compare the efficacy and acceptability of panretinal photocoagulation (PRP) associated with intravitreal conbercept injections versus PRP alone in the treatment of patients with PDR.

**Methods:**

The Preferred Reporting Items for Systematic Reviews and Meta-Analyses Protocols reporting guidelines and the recommendations of the Cochrane Collaboration were followed to conduct this study. Reviewers will search the PubMed, Cochrane Library, Web of Science, and EMBASE online databases using the key phrases “panretinal photocoagulation,” “conbercept,” and “proliferative diabetic retinopathy” for all cohort studies published up to May 2021. The studies on cohort study focusing on PRP + conbercept and PRP alone for PDR patients will be included in our meta-analysis. At least one of the following outcomes should have been measured: PRP completion rate, proportion of eyes with visual gain/loss, central macular thickness, and incidence of complication. Review Manager software (v 5.4; Cochrane Collaboration) is used for the meta-analysis.

**Results:**

It was hypothesized that intravitreal conbercept plus PRP was more effective than PRP alone.

**OSF registration number:**

10.17605/OSF.IO/HCQ2S.

## 1 Introduction

Proliferative diabetic retinopathy (PDR) is the leading cause of blindness in working-age adults in the United States, with 12,000 to 24,000 new cases each year. Panretinal photocoagulation (PRP) has been the standard treatment for PDR since the Diabetic Retinopathy Study demonstrated its benefit nearly 40 years ago.^[1]^ PRP is effective in part because it reduces vascular endothelial growth factor (VEGF). In a 2014 research, 98% of retinal specialists reported using PRP for initial PDR management in the absence of diabetic macular edema.^[2]^ However, PRP may lead to permanent peripheral vision loss and loss of night vision, and may exacerbate diabetic macular edema, making alternative therapy desirable. Even with timely PRP treatment, about 5% of PDR eyes will experience severe vision loss.^[3]^

More recently, intravitreal injections of conbercept and other anti-VEGF agents have been administered several days before patients undergo vitrectomy to improve surgical efficiency and reduce surgical complications.^[4,5]^ The beneficial effect of conbercept as an adjuvant to vitrectomy is attributed to its multiple targets on the VEGF family of factors, which inhibit the growth of new blood vessels and reduce the vascular permeability of the retina.^[6,7]^ Although conbercept has been used for other diseases associated with new vascular formation, timing changes in retinal neovascularization response to PRP alone or single-dose conbercept in combination with PRP have not been established.^[8]^

Therefore, in order to provide new evidence-based medical evidence for clinical treatment, we conducted this protocol for systematic review and meta-analysis to compare the efficacy and acceptability of PRP associated with intravitreal conbercept injections versus PRP alone in the treatment of patients with PDR. It was hypothesized that intravitreal conbercept plus PRP was more effective than PRP alone.

## 2 Materials and methods

### 2.1 Search strategy

The Preferred Reporting Items for Systematic Reviews and Meta-Analyses Protocols reporting guidelines and the recommendations of the Cochrane Collaboration were followed to conduct this study. Reviewers will search the PubMed, Cochrane Library, Web of Science, and EMBASE online databases using the key phrases “panretinal photocoagulation,” “conbercept,” and “proliferative diabetic retinopathy” for all cohort studies published up to May 2021. There is no restriction in the dates of publication or language in the search for the current review, and thus publication and language bias can be minimized. Ethical approval is not necessary because the present meta-analysis will be performed based on previous published studies. The prospective registration has been approved by the Open Science Framework registries (with the number 10.17605/OSF.IO/HCQ2S).

### 2.2 Eligibility criteria

The studies on cohort study focusing on PRP + conbercept and PRP alone for PDR patients will be included in our meta-analysis. At least one of the following outcomes should have been measured: PRP completion rate, proportion of eyes with visual gain/loss, central macular thickness, and incidence of complication. The exclusion criteria contains biochemical trials, reviews, case reports, no assessment of outcomes mentioned above, and no comparison of PRP + conbercept and PRP alone for PDR patients.

### 2.3 Data extraction

A standard data extraction form is used independently by 2 reviewers to retrieve the relevant data from the articles. These variables include author, study design, sample size, publishing date, population, type of interventions, type of controls, follow-up, and outcomes. The outcome measures are as following: PRP completion rate, proportion of eyes with visual gain/loss, central macular thickness, and incidence of complication. Data extraction is performed independently, and any conflict is resolved before final analysis. If data are not presented in the original article, corresponding authors will be contacted to acquire the missing data. Otherwise, the results are extracted manually from the published figures. If necessary, we will abandon the extraction of incomplete data.

### 2.4 Data analysis

Review Manager software (v 5.4; Cochrane Collaboration) is used for the meta-analysis. Extracted data are entered into Review Manager by the first independent author and checked by the second independent author. Risk ratio with a 95% confidence interval or standardized mean difference with 95% CI are assessed for dichotomous outcomes or continuous outcomes, respectively. The heterogeneity is assessed by using the *Q* test and *I*^2^ statistic. An *I*^2^ value of <25% is chosen to represent low heterogeneity and an *I*^2^ value of >75% to indicate high heterogeneity. All outcomes are pooled on random-effect model. A *P* value of <.05 is considered to be statistically significant.

### 2.5 Quality assessment

The quality of randomized trials will be assessed by Cochrane risk of bias tool for randomized controlled trials and the risk of bias in nonrandomized studies - of interventions for nonrandomized, observational studies. Each paper will be reviewed by 1 reviewer and verified by a second and disagreements will be resolved by discussion with a third reviewer. A meta-analysis will be conducted when 3 or more trials reported an outcome of interest. We also will perform the sensitivity analysis to evaluate whether the differences of study design had an impact on the overall estimate and data. Review Manager software (v 5.4; Cochrane Collaboration) will be conducted for statistical investigation and a funnel plot analysis will be drawn to assess the publication bias if there are more than 10 studies included.

## 3 Discussion

PDR is the leading cause of severe vision loss in patients with diabetes worldwide, and is characterized by retinal neovascularization at the disc or elsewhere in the retina. Conbercept was made available for clinical application as an inhibitor of the VEGF-A, VEGF-B, and placental growth factor receptors. Although conbercept has been used for other diseases associated with new vascular formation, timing changes in retinal neovascularization response to PRP alone or single-dose conbercept in combination with PRP have not been established. Therefore, in order to provide new evidence-based medical evidence for clinical treatment, we conducted this protocol for systematic review and meta-analysis to compare the efficacy and acceptability of PRP associated with intravitreal conbercept injections versus PRP alone in the treatment of patients with PDR. It was hypothesized that intravitreal conbercept plus PRP was more effective than PRP alone.

## Author contributions

**Conceptualization:** Zhaoli Chen.

**Data curation:** Liangyu Wang, Zhaoli Chen.

**Formal analysis:** Liangyu Wang, Zhaoli Chen.

**Funding acquisition:** Xiaoxue Wang.

**Investigation:** Liangyu Wang, Zhaoli Chen.

**Methodology:** Zhaoli Chen.

**Project administration:** Xiaoxue Wang.

**Resources:** Liangyu Wang, Zhaoli Chen, Xiaoxue Wang.

**Supervision:** Xiaoxue Wang.

**Validation:** Xiaoxue Wang.

**Writing – original draft:** Liangyu Wang.

**Writing – review & editing:** Xiaoxue Wang.
